# Comprehensive search for intra- and inter-specific sequence polymorphisms among coding envelope genes of retroviral origin found in the human genome: genes and pseudogenes

**DOI:** 10.1186/1471-2164-6-117

**Published:** 2005-09-09

**Authors:** Nathalie de Parseval, Gora Diop, Sandra Blaise, François Helle, Alexandre Vasilescu, Fumihiko Matsuda, Thierry Heidmann

**Affiliations:** 1 UMR 8122 CNRS, Institut Gustave Roussy, 39 rue Camille Desmoulins, 94805 Villejuif Cedex, France; 2 Centre National de Génotypage, 2, rue Gaston Crémieux, Évry, France

## Abstract

**Background:**

The human genome carries a high load of proviral-like sequences, called Human Endogenous Retroviruses (HERVs), which are the genomic traces of ancient infections by active retroviruses. These elements are in most cases defective, but open reading frames can still be found for the retroviral envelope gene, with sixteen such genes identified so far. Several of them are conserved during primate evolution, having possibly been co-opted by their host for a physiological role.

**Results:**

To characterize further their status, we presently sequenced 12 of these genes from a panel of 91 Caucasian individuals. Genomic analyses reveal strong sequence conservation (only two non synonymous Single Nucleotide Polymorphisms [SNPs]) for the two HERV-W and HERV-FRD envelope genes, i.e. for the two genes specifically expressed in the placenta and possibly involved in syncytiotrophoblast formation. We further show – using an *ex vivo *fusion assay for each allelic form – that none of these SNPs impairs the fusogenic function. The other envelope proteins disclose variable polymorphisms, with the occurrence of a stop codon and/or frameshift for most – but not all – of them. Moreover, the sequence conservation analysis of the orthologous genes that can be found in primates shows that three *env *genes have been maintained in a fully coding state throughout evolution including *env*W and *env*FRD.

**Conclusion:**

Altogether, the present study strongly suggests that some but not all envelope encoding sequences are *bona fide *genes. It also provides new tools to elucidate the possible role of endogenous envelope proteins as susceptibility factors in a number of pathologies where HERVs have been suspected to be involved.

## Background

A large fraction (8%) of the human genome contains elements of retroviral origin, with thousands of sequences closely related to the integrated proviral form of infectious retroviruses with the canonical *gag*, *prt*, *pol *and *env *genes [1]. These elements, named human endogenous retroviruses (HERV), are most probably the proviral remnants of ancestral germline infections by active retroviruses, which have thereafter been transmitted in a Mendelian manner. HERVs have been grouped according to sequence homologies into more than 80 distinct families, each containing a few to several hundreds elements (reviewed in [2-4], see [5] for their classification). Most HERV genes are non-coding, due to either terminating mutations or deletions, but we have characterized 16 human endogenous *env *genes that have retained a coding capacity among the 30,000 endogenous proviral elements of the human genome [6]. The analysis of their transcriptome in healthy human tissues has revealed that three of them are highly expressed in the placenta, namely the erv3/HERV-R, the HERV-W and the HERV-FRD *env *genes [6].

Phylogenetic as well as functional analyses have revealed strong similarities between HERVs and the present-day infectious retroviruses, suggesting a common history and shared ancestors. Accordingly, it has been proposed that HERVs may still possess some of the functions of infectious retroviruses and as such have pathogenic effects, provided that they are transcriptionally active. Conversely, it is also plausible that HERV proteins may have been co-opted by the host for its benefit. Along this line, it has been proposed that the HERV envelope proteins could play a role in several processes including i) protection against infection by present-day retroviruses through receptor interference [7], ii) protection of the fetus against the maternal immune system via an immunosuppressive domain located in the envelope transmembrane (TM) subunit [8,9], and iii) placenta morphogenesis through fusogenic effects allowing differentiation of cytotrophoblastic cells into the syncytiotrophoblast [10-12]. In accordance with a symbiotic role for HERVs, it has recently been shown that the HERV-W and HERV-FRD envelope gene products are highly fusogenic glycoproteins that are specifically expressed in the placenta and can mediate cell-cell fusion *ex vivo *[12,13]. Involvement of HERV proteins in physiological processes, however, remains a debated issue, and definite evidence is still lacking. Because selection pressure on a functional gene should result in a limited mutation rate, the survey of single nucleotide polymorphisms (SNPs) among the human population is a way to evaluate functional constraints on these genes. Using this approach, we had previously demonstrated that one postulated candidate for a role in placentation, namely the highly-expressed erv3/HERV-R envelope gene carries a homozygous stop mutation resulting in a severe protein truncation in 1% of individuals of caucasian origin, which strongly suggests that it is not necessary for any fundamental placental function [14]. The unexpectedly low number of still coding envelope genes present in the human genome [6] now allows a comprehensive analysis of such genes to be performed, in order to assess their possible physiological and/or physiopathological role. Here, we analysed the SNP level of the 12 coding *env *genes present in the human genome that could be characterized by this approach, together with their conservation among the orthologous genes that can be identified in primates. The two series of data are consistent with a role beneficial to the host for some of the genes, whereas others are likely to be subjected to progressive inactivation. In both cases, the identified SNPs should be useful tools to evaluate the possible role of these genes as "susceptibility genes" in several human pathologies where HERVs have been suspected to be involved.

## Results

### Structure and PCR-amplification of the fully coding HERV envelope genes

**Figure 1:**
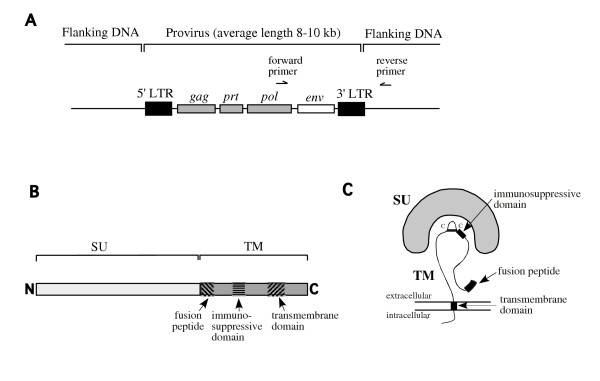
**Schematic representation of the proviral form of a retrovirus and its *env *gene products**. A, Genomic structure of a provirus, with the four canonical retroviral genes *gag *(encoding the virion core proteins), *prt *(encoding the protease), *pol *(encoding the reverse transcriptase, RNAseH, integrase), and *env *(encoding the viral membrane protein). The position of the primers designed to amplify the coding *env *gene is indicated. B, Linear representation of a retroviral envelope protein and its functional domains. The *env *gene encodes a polypeptide which is cleaved into two proteins, the surface protein (SU), which is involved in receptor recognition, and the transmembrane subunit (TM), which anchors the whole envelope complex to the membrane and is directly responsible for cell membrane fusion and virus entry. The TM subunit contains three functional domains, the fusion peptide, the immunosuppressive domain and the transmembrane domain. C, Schematic structure of the envelope gene products. C-C: disulfide bound.

Retroviral envelope genes are 2 kb-long sequences with no introns, that are located in the 3' domain of proviral elements (see Figure 1A for the genomic structure of a provirus and Figure 1B and 1C for the description of the envelope gene and its product). Endogenous retroviruses being in most cases highly reiterated elements (see Table 1), the 3' PCR primers for *env *gene amplification had to be placed downstream of the provirus end to specifically amplify the family member of interest, whereas the 5' primer was placed upstream of the *env *MET initiation codon (see Figure 1A). Among the 16 coding *env *genes that we had previously identified [6], 12 could be subjected to a systematic search for SNPs, including 3 out of the 6 HERV-K(HML-2) coding *env *genes (K1, K2, K4), 2 out of the 3 HERV-H coding *env *genes (H1 and H3), and the coding *env *genes of the F(c)1, F(c)2, T, W, R, R(b) and FRD families. Three HERV-K(HML-2) *env *genes could not be PCR amplified: the first one (*env*K3) is located in the centromeric region of chromosome 19, and its provirus is surrounded by stretches of repeated sequences, thus precluding the use of specific primers; the other two (*env*K5 and *env*K6) belong to proviruses present only in a fraction of the caucasian population [15], with a low allele frequency (0.19 and 0.04, respectively), thus precluding a statistically significant SNP study to be performed (unless a pre-selected population was used). It is noteworthy that proviruses K1, K2 and K4 have also been recently demonstrated to be polymorphic, since they exist in some individuals as a solo Long Terminal Repeat (LTR), devoid of internal sequences [16]. Yet, the allele frequencies of the complete provirus forms are high (0.72, 0.97 and 0.89, respectively), thus allowing an SNP study. Finally, sequence data for one of the 3 HERV-H coding *env *genes studied (i.e. *env*H2) yielded multiple sequence profiles (possibly due to the parasitic amplification of another member of the HERV-H family) and could not be analyzed further. The list of the 12 fully coding *env *genes that could finally be analyzed, together with the associated amplification primers used, is given in Table 1.

**Table 1:** The coding envelope genes of the human genome studied for their SNPs.

Gene name	Bibliographic gene name	Family name (repbase identifier)	Approximate number of elements	Genomic localization	Amplification primers sequence (5'-3')
*env*K1	-	HERV-K(HML-2) (HERVK)	50–100	Chr12: 57008431–57010527 (-)	F: GGGAAATAGGGAAGGTGATA R: ACATCCCTAACGCTTTAAG
*env*K2	HML-2.HOM, HERV-K108	idem	idem	Chr7: 4367317–4369416 (-)	F: GAGGTTTTGCTTGTGTTTCA R: TTAGGCTTTCGGGACTTCAA
*env*K4	HERV-K109	idem	idem	Chr6: 78423172–78425268 (-)	F: GGGAAATAGGGAAGGTGATA R: GGGTAGTATCAGTCGGGATG
*env*F(c)1	-	HERV-F(c)1 (-)	1	Chr:X: 95874118–95875872 (+)	F: GCACCGACTCAGCACGAC R: GCCTTGGCAACTAAACCATTC
*env*F(c)2	-	HERV-F(c)2 (-)	15	Chr7:152498167–152499936 (-)	F: GAAGGCACCTACACAACATC R: GACACTTAATAGTTGCGACA
*env*H1	envH/p62, H19	HERV-H (HERVH)	1000	Chr2: 166767244–166768998 (-)	F: ATGCCCTACTCTTGTTTACAC R: AAATCTGGCAAACTACAAGC
*env*H3	envH/p59	idem	idem	Chr2: 155931277–155932944 (+)	F: TTTCTTCAAGCCATCACAGC R: ACCCCATGTTCTAGTCTTCC
*env*T	-	HERV-T (HERVS71)	50	Chr19: 20341241–20343121 (+)	F: TTGGATTCATCACTCCCA R: CTGAAGGGAGTTCCTCCTAGG
*env*W	Syncytin 1	HERV-W (HERV17)	200	Chr7: 91710108–91711724 (-)	F: AACAACCAGGAGGAAAGTAA R: CTGATCAAGTCGCAAAGC
*env*R	erv3	HERV-R (HERV3)	100	Chr7: 63863079–63865094 (-)	F: GGTTAGAAATCTGAAGTCC R: AAAGTCAATGACAGATGCGG
*env*R(b)	-	HERV-R(b) (PABL_B)	50	Chr3: 16786814–16788358 (+)	F: GCTAAGCACCAGTTCAGCACTG R: TGTTTTGGGACACCACGAAT
*env*FRD	Syncytin 2	HERV-FRD (MER50)	3000	Chr:6: 11211913–11213529 (-)	F: CTTGTACACCACCAGGAGTTCC R: TTTGAGCAAGGGTGATTCAT

### SNP of the human coding envelope genes and haplotype analysis

**Figure 2:**
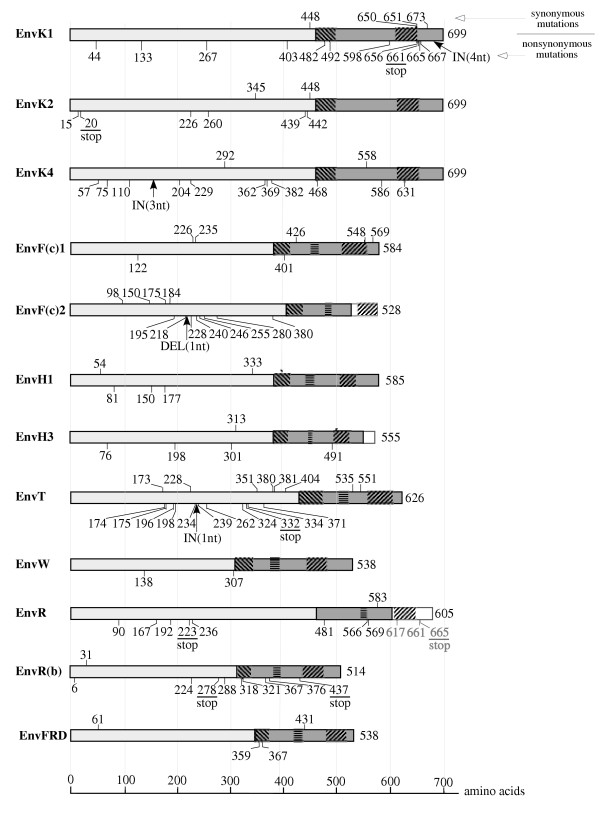
**Localization of the SNPs on the envelope genes**. Characteristic domains of the envelope proteins are depicted as in Figure 1B. The gray frames at the end of H3, R and F(c)2 genes represent short open reading frames present downstream of the stop codon. Mutations are represented along the protein sequence, with the number corresponding to the amino acid affected by the SNP. Synonymous mutations are indicated above the protein frame. Non-synonymous (with stop mutations indicated) and indel mutations (with frame shifting mutations underlined) are indicated below the protein frame.

**Figure 3:**
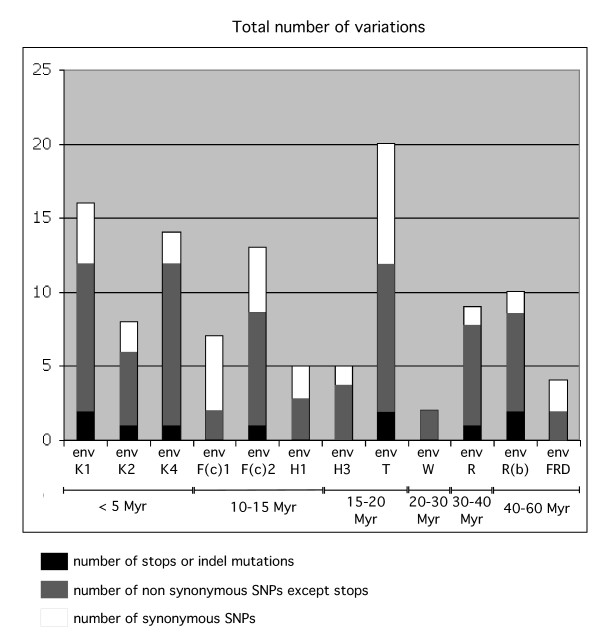
**Number of variations identified in the HERV coding envelope genes**. The SNP numbers are indicated for each *env *gene, with the number of synonymous SNPs, nonsynonymous SNPs except stops, and stops or indel mutations indicated. The *env *genes are ordered according to their date of entry in the primate lineage (see [4]), indicated in million years (Myr).

The 12 HERV coding *env *genes were PCR-amplified for 91 healthy Caucasian individuals, and each PCR product was directly sequenced without cloning. The identified SNPs are positioned on the protein sequences in Figure 2, and the number and nature of the variations (synonymous [i.e. silent] versus nonsynonymous [i.e. leading to an amino acid change] substitutions, stop or insertion/deletion [indel] mutations) are given in Figure 3. The complete list and detailed features – including frequencies – of the identified SNPs is available in additional data file 1. As illustrated in Figure 2 and 3, the number of SNPs for the 12 *env *genes varies significantly depending on the gene, but their distribution along the envelope sequences seems to be random. The number of SNPs per kb varies from 1 (for *env*W) to 10 (for *env*T). Studies on SNPs in intragenic regions of the human genome disclose average densities varying from 0.8 to 5 SNPs per kb [17,18]. These studies also provide an estimate of the average ratio of synonymous versus non-synonymous SNPs, which is close to 1 in coding regions, whereas it is close to 0.5 in pseudogenes [17,18]. For the *env *genes, provided that the SNP number is high enough to validate this ratio (thus excluding *env*FRD, *env*W, *env*F(c)1, *env*H1 and *env*H3), it ranges from 0.11 for *env*R(b) to 0.66 for *env*T, close to the pseudogene ratio.

Based on the number of nonsynonymous and indel mutations, a hierarchy among the endogenous coding *env *genes can be established, with the *env*W, *env*FRD and *env*F(c)1 genes being the most conserved (see also [19]), and the *env*K1, *env*K2, *env*T, *env*R, *env*R(b), and *env*F(c)2 genes being affected by numerous mutations, including mutations resulting in truncation of the protein due to frameshifting or generation of stop codons. There is no correlation between the number of SNPs and the "age" of the corresponding gene in the primate lineage. This is clearly illustrated in Figure 3, where the *env *genes have been ordered according to the date of entry of each corresponding provirus into the host genome as previously determined via an analysis of the orthologous loci throughout evolution (reviewed in [4]). For instance, the *env *genes of the HERV-K(HML-2) family are human-specific, i.e. are present in the genome of primate since less than five million years (Myrs), whereas *env*R(b) and *env*FRD have entered the genome of the common ancestor of Old World and New World monkeys more than 40 Myrs ago. For the *env*FRD gene, which is among the "oldest" *env *genes, only 4 SNPs are found, whereas for the "recent" *env *genes of the HERV-K(HML-2) family, the SNP number can be as high as 16. Although the lack of correlation between the "age" of the genes in the primate lineage and the numbers of SNPs is not unexpected taking into account the occurrence of "bottlenecks" giving rise to founder effects during the evolution of the human population, what remains surprising is the important variability of the number and "severity" of SNPs among the *env *genes. This should be a strong indication for a differential selection pressure exerted on these genes (see below).

A further characterization of the SNPs, including genotype distribution, haplotype frequency, and linkage disequilibrium was performed (additional data files 1, 2 and 3). The genotype distributions are compatible with a Hardy-Weinberg equilibrium, except for some positions on the HERV-K1 (HERV-K1_44, HERV-K1_133, HERV-K1_403, HERV-K1_482, HERV-K1_651, HERV-K1_673, HERV-K1_2144), HERV-K4 (HERV-K4_292, HERV-K4_369, HERV-K4_382, HERV-K4_586) and HERV-F(c)1 (HERV-F(c)1_122, HERV-F(c)1_226, HERV-F(c)1_235) *env *genes, consistent with recent integration of these elements in the primate lineage (see Figure 3). Haplotype frequencies were estimated for each gene based on the Expectation-Maximization (EM) algorithm [20] for haplotypes with frequency estimates >1%. The results are summarized in additional data file 2. The three most frequent haplotypes for each *env *gene represent >80% of all the haplotypes, suggesting that these regions have a low recombination rate. A linkage disequilibrium (LD) plot was generated (additional data file 3) with pairwise LDs measured between each pair of polymorphisms using the D and D' methods (see materials and methods). As expected, the majority of high LD values occur within the *env *genes, and the LDs calculated between SNPs of different HERV *env *genes is low. Low LDs were obtained as well for *env *genes located on the same chromosome, i.e. for HERV-F(c)2, HERV-K2, HERV-R, HERV-W (on chromosome 7) and for HERV-H1 and HERV-H3 (on chromosome 2; 10 Mb apart). These observations suggest an independent evolution for each *env *gene.

**Figure 4:**
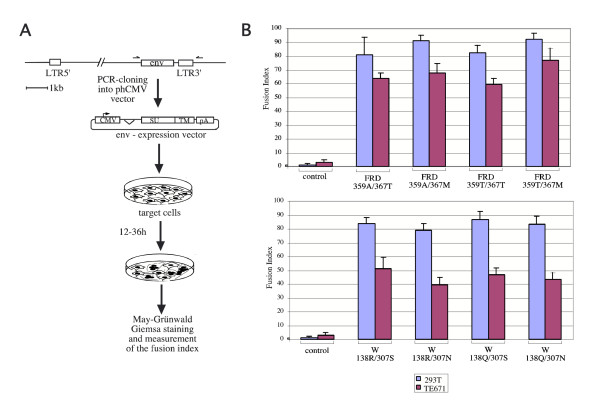
**Effects of the non synonymous SNPs identified in envFRD and envW on their fusogenic function**. A, Construction of the env-expression vectors and rationale of the fusion assay. Each of the *env *allelic forms were PCR amplified from genomic DNA and cloned into the phCMV expression vector. Cells were transfected with the env-expression vectors and stained with May-Grünwald and Giemsa solutions. B, Cell-cell fusion assay for the allelic forms of envFRD (upper panel) and envW (lower panel), using two cells types (human 293T and TE671 cells). The fusion index represents the percentage of fusion events in the transfected cell populations as evidenced by syncytia formation, and is quantitated as in [12]. The control corresponds to transfection of an expression vector without an *env *gene. The most frequent haplotypes correspond to FRD359A/367T and W138R/307S.

Among all the coding *env *genes, *env*W and *env*FRD are the only two genes with a clearly identified functional property, i.e. the capacity to generate cell-cell fusion [10,12]. This property – most probably associated with its role in placentation, see the Background section – was used to characterize further the consequences of the identified SNPs on the fusogenic function of the encoded proteins. To do so, we PCR-amplified the genomic DNAs of individuals carrying the corresponding SNP alleles, with primers allowing the cloning of the *env *genes in appropriate expression vectors. The fusogenic function was then assayed as in [12], using two different cell lines for fusion (Figure 4). As illustrated in the figure, no difference can be observed between the four haplotypes of each of the *env *genes tested. This, together with the low SNP level for the two genes, is a strong indication for selection of a "function" associated with the corresponding proteins.

### Interspecific sequence conservation

**Figure 5:**
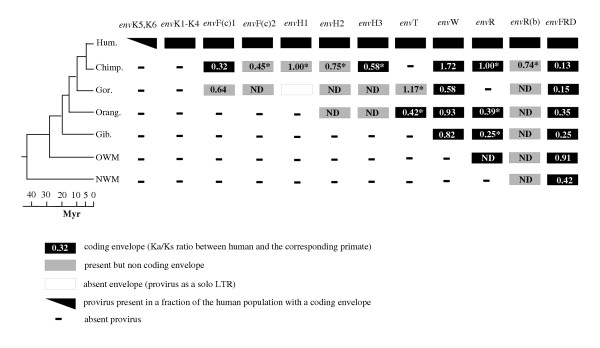
**Conservation of the coding envelopes in primate species**. Hum.: human, Chimp.: chimpanzee, Gor.: gorilla, Orang.: orangutan, Gib.: gibbon, OWM: Old World Monkeys, NWM: New World Monkeys. The boxes indicate presence of the *env *gene, with its coding status illustrated with a color code.

The data regarding the orthologous genes that can be found in primates for each of the presently studied human *env *genes are given in Figure 5. Some of the orthologous primate *env *genes had been previously cloned and sequenced ([19] for *env*W, [21] for *env*FRD, [22] for *env*R and [23] for envF(c)1). Others have been PCR-amplified and tested using a direct coupled *in vitro *transcription/translation assay to determine their coding status ([24] for the 3 *env*H and this study for envT, envF(c)2 and envR(b), see Materials and Methods). As illustrated in the figure, a first important outcome is that only three *env *genes have been maintained in a fully coding state throughout evolution, i.e. *env*FRD (7/7 lineages), *env*W (5/5 lineages) and *env*R (5/5 lineages) which, interestingly, correspond to the three *env *genes highly expressed in the placenta. Secondly, as observed for the human SNP analysis, there is no correlation between the "age" of the gene in the primate lineage and its coding status: the latter three *env *genes are among the "oldest" ones, whereas three other *env *genes (*env*H3, *env*T and *env*F(c)1) are fully coding in only one non-human lineage, and the others are only coding in the human lineage.

## Discussion

The present investigation of the fully coding human *env *genes, including the human SNP search and the analysis of the coding status of the identified primate orthologs, pinpoints two of these genes – namely *env*FRD and *env*W – that disclose the characteristic features of a gene subjected to a functional constraint, i.e. low polymorphism and maintenance of an open reading frame during evolution. Interestingly, these two genes are highly – and specifically – expressed in the placenta, and possess a well-characterized fusogenic function which led to the proposal that they are *bona fide *genes that have been co-opted by the host for a physiological function related to placenta physiology [10,12,13,19]. Among the other genes, *env*R is of interest since as with the two former genes it is highly expressed in the placenta and has maintained its fully coding status in all species since its entry into the primate lineage. Yet, the SNP analysis discloses a severe polymorphism – including a premature stop codon – indicating that the preservation of an open reading frame during evolution should not be considered as a sufficient criterion to assign a biological function. The other genes are not conserved in a fully coding state in all the primate branches where they are present and/or disclose a severe polymorphism (with in several cases occurrence of a premature stop codon for some allelic forms).

Another hallmark of the presence of a functional constraint on a gene is a low nonsynonymous/synonymous substitution ratio (Ka/Ks) for orthologous genes found in other species. A *bona fide *gene with a cellular function should be under purifying selection, which prevents deleterious nonsynonymous substitutions from being fixed and usually does not affect synonymous substitutions, leading to Ka/Ks ratios <1, whereas a gene under genetic drift (e.g. a pseudogene) has a Ka/Ks ratio close to unity (reviewed in [25]). Such an analysis has already been performed for the *env*FRD and the *env*W genes. The mean ratio for all pairwise comparisons was 0.37 for *env*FRD [21], demonstrating the existence of a selective pressure. For *env*W, the corresponding ratio was 0.8 [19], precluding definite conclusions (yet, a subsequent study on *env*W identified a region of the gene with a lower ratio compatible with a functional constraint specifically exerted on that domain [26]). We have calculated the Ka/Ks ratios for the other *env *genes, when nucleotide sequences of their orthologs were available (data not shown). These ratios were found to be heterogeneous for a given *env *gene, with values ranging from 0.23 to 1.17, again precluding any definite conclusion to be drawn.

According to the present analysis of intra- and interspecific variability of the *env *genes, one is led to conclude that most probably only two among the twelve studied *env *genes are likely to be involved in an essential human physiological function, whereas the others would be on their way to conversion into pseudogenes. The presence of a reading frame still open in human for the latter genes may appear intriguing, but one has to keep in mind that they belong to multicopy HERV families, and as such one of the element could have remained open just by chance, without any purifying selection, since even under completely neutral drift it takes time for a sufficient number of mutations to transform a gene into a pseudogene. Along this line, it is of interest to mention the study by Zhang and Webb on the primate *V1R *pheromone receptor genes, for which there were approximately 140 copies in the genome of the common ancestor of Old World monkeys and hominoids, whereas the human genome has only five *V1R *genes that retained an ORF. Examination of the orthologous genes in primates showed that none of the five genes kept an intact ORF in all of the apes. Furthermore, for the orthologous sequences with an intact ORF, Ka/Ks ratios were close to unity. The intraspecific variation of these five human genes was also assessed, and for two of them an allelic form generating a premature stop codon was found. Altogether, the authors concluded that there were no functional constraints on these genes since before the separation of hominoids and Old World Monkeys (approximately 23 Myrs ago) and that they were in the process of pseudogeneization in those primate species [27]. Another possible explanation for the "neutral" conservation of an open reading frame for an HERV *env *gene without any selection pressure from the host could be related to the relatively autonomous status of these parasitic elements, and be associated with the persistence of active retroviral elements responsible for the maintenance – by a reiterated infection process – of some of the HERV families (e.g. [23,28]).

In any case, it appears clearly that conservation of an *env *gene with a coding status cannot be taken as the sole criterion for a possible function to the benefit of the host, with only the *env*W and *env*FRD genes emerging from the present study as possible *bona fide *genes. Yet, this does not mean that the other *env *genes cannot have any effect in humans. Indeed, the present analysis only indicates that they are not under stringent purifying selection, in terms of evolution, but they still could be involved in pathologies – such as tumors or auto-immune diseases – not deleterious to the species because they occur late in the life of the individuals. One should keep in mind that endogenous retroviruses originate from *bona fide *retroviruses, and as such might have conserved some of the pathological potency of their progenitors. In this respect, the identified SNPs should be essential tools to determine if this is indeed the case, via an analysis of their distribution among selected groups of individuals with a definite pathology. Along this line, the present data on the *env*T gene are of special interest. Indeed, this gene is the only non-placental *env *gene found to be highly expressed in a human tissue – the thyroid – of healthy individuals [6], and the high level of polymorphism of the gene shown in this report together with its lack of conservation in primates suggest that it is not involved in any essential physiological process and thus not subjected to purifying selection. Thanks to the identified SNPs, it can now be tested whether this expressed gene is involved in a pathological process in humans, among which thyroid tumors could be select candidates for a systematic search.

## Conclusion

The systematic SNP search on fully coding human endogenous envelope genes, combined with an analysis of the sequence conservation among the orthologs that can be identified among primates revealed that two genes (*env*W and *env*FRD) can be considered as *bona fide *genes, and identified polymorphisms – to a variable extent – in the other genes. The data are consistent with a physiological role for the former (also called syncytin-1 and syncytin-2 and likely to be involved in human placentation) and provide tools for the latter, to determine their potential role in physiological processes and/or their association with pathological processes in humans – which would be the consequence of their original retroviral status.

## Methods

### DNA samples and genotyping

Ninety-one human samples of French Caucasians were collected from the EGEA (Epidemiological study on the Genetics and Environment of Asthma) study, among the controls ascertained without disease. PCR was performed in mixture containing 25 ng of DNA, 0.3 pmol of each primer, 6 nmol of each dNTP, 0.75 units of ExTaq and 1× reaction buffer (Takara). Sequencing reactions were performed according to the Dye Terminator method using an ABI PRISM^® ^3700 DNA Analyzer (Applied Biosystems, Foster City, CA, USA). Alignment of sequences, SNP discovery and genotyping were performed with *Genalys *software [29]. The genomic sequences used for the alignment are *env*FRD (GenBank accession no. AL136139), *env*R (AC073210), *env*T (AC078899), *env*W (AC000064), *env*Fc(1) (AL354685), *env*Fc(2) (AC01222), *env*H1 (AJ289709), *env*H2 (AJ289710), *env*H3 (AJ289711), *env*R(b) (AC093488), *env*K1 (AC074261), *env*K2 (AC072054), *env*K4 (AF164615). The sources of the simian genomic DNAs are given in ref [24].

### Statistical analysis

The haplotypes frequencies using all polymorphisms for each gene were estimated with the EM Algorithm [20]. The linkage disequilibrium (LD) estimates between pairs of polymorphisms were obtained by estimating the two polymorphisms haplotypes frequencies using this algorithm. Computation of D and D' (standard disequilibrium measure and standardized disequilibrium measure) in additional data file 3 was performed as in ref [30].

### Cloning of allelic forms of the W and FRD human *env *genes in expression vectors

The FRD and W *env *genes were PCR-amplified from human genomic DNAs. PCR was carried out for 25 cycles (10 sec at 93°C, 30 sec at 56°C, 4 min at 68°C), in 50 μl, using 100 ng of genomic DNA, 48 pmol of each primer, 350 μM of each dNTP, 0.75 μl Expand long template enzyme mix and 1× reaction buffer (Roche Applied Science). For the FRD *env *gene, *Xho*I-containing primers were ATCACCTCGAGCACCATGGGCCTGCTCCTGCTGGTTCTCATTC as forward primer and ATCACCTCGAGGCTTCAGTACAGGTGGATA as reverse primer. For the W *env *gene, *Xho*I-containing primers were ATCACCTCGAGAACAACCAGGAGGAAAGTAA as forward primer and ATCACCTCGAGCTGATCAAGTCGCAAAGC as reverse primer. Each PCR product was then *Xho*I-cleaved and cloned into the phCMV-G vector (described in [12]) opened with *Xho*I. Allelic forms of the cloned *env *genes were assessed by enzymatic restriction. For *env*FRD, the 1075 G->A transition (aa 359) predicts the loss of a RsaI site and the 1100 C->T transition (aa 367) predicts the loss of a BstUI site, and for *env*W the 413 G->A transition (aa 138) predicts the gain of a BstXI site and the 920 G->A transition (aa 307) predicts the gain of a Tsp509I site. As two allelic forms (FRD359T/367M and W138Q/307S) were not available among the cloned envelope genes, we constructed them by exchange of restriction fragments (BsmI-NotI for FRD359T/367M and KpnI-XhoI for W138Q/307S).

### Cell-cell fusion assay

The human TE671 rhabdomyosarcoma cells (ATCC CRL8805) and 293T embryonal kidney cells (ATCC CRL11268) were grown in Dulbeco's modified Eagle's medium (DMEM) supplemented with 10% fetal calf serum. All cell culture media were supplemented with streptomycin (100 μg/mL) and penicillin (100 U/mL). Cells were transfected using calcium phosphate precipitation (Invitrogen, 5 μg of DNA for 5 × 10^5 ^cells). Fusion activity of envelope glycoproteins was measured 12 to 36 h after transfection of the cells with the corresponding expression vectors. To visualize syncytia, cells were fixed in methanol and stained by adding May-Grünwald and Giemsa solutions (Sigma) according to the manufacturer's instructions. The fusion index, which represents the percentage of fusion events in a cell population is defined as [(N-S)/T] × 100, where N is the number of nuclei in the syncytia, S is the number of syncytia, and T is the total number of nuclei counted.

### Characterization of the orthologous *env*T, envF(c)2 and *env*R(b) *env *gene ORFs from simians

The size of the *env *gene open reading frame in the primate loci was evaluated using a direct coupled *in vitro *transcription/translation assay based on T7 promoter-containing PCR products as described in [24], which allows to determine the status of both alleles in the same assay.

For the amplification of *env*T from gorilla and orangutan, the forward T7 promoter-containing primers were GCTAATACGACTCACTATAGGAACAGACCACCATGTCCTGCTTGGATTCATCAC and GCTAATACGACTCACTATAGGAACAGACCACCATGTTGGATTCATCACTCCCA, respectively, and the common reverse flanking primer was CTGAAGGGAGTTCCTCCTAGG. For the amplification of *env*R(b) from chimpanzee, gorilla, orangutan, gibbon, Rhesus macaque (Old World Monkey) and *Callithrix jacchus *(New World Monkey) the forward T7 promoter-containing primer was GCTAATACGACTCACTATAGGAACAGACCACCATGGATCCACTACACACGATTGA and the reverse flanking primer was TGTTTTGGGACACCACGAAT. For the amplification of *env*F(c)2 from chimpanzee and gorilla, the forward T7 promoter-containing primer was GCTAATACGACTCACTATAGGAACAGACCACCATGAATTCTCCATGTGAC and the reverse flanking primer was GACACTTAATAGTTGCGACA.

The simian *env *gene sequences were deposited in Genbank with accession numbers AJ862646-AJ862655.

## List of abbreviations used

HERV, human endogenous retrovirus; TM, transmembrane; SNP, single nucleotide polymorphism; LTR, Long Terminal Repeat; LD, linkage disequilibrium.

## Authors' contributions

NdP designed the cloning primers for the *env *genes, carried out the interspecific sequence conservation studies and drafted the manuscript.

GD carried out the SNP studies.

SB carried out the cloning of the *env *genes and the cell-cell fusion assays.

FH participated in the interspecific sequence conservation studies.

AV designed the sequencing primers, participated in the coordination of the SNP studies and in drafting the manuscript.

FM coordinated the SNP studies.

TH conceived the study.

## Supplementary Material

Additional data file 1 Additional data file 1 is a table listing the polymorphism of HERV coding envelope genes. Click here for file

Additional data file 2 Additional data file 2 is a figure showing polymorphism combinations and estimated frequencies for haplotypes in the 12 HERV coding *env *genes. The SNPs are identified by their CNG ID (see additional data file 1). Click here for file

Additional data file 3 Additional data file 3 is a figure showing the LD plot of 12 HERV coding *env *genes. The LD pattern is shown with the D values above and the D' values below the diagonal, and estimated allele frequencies for each polymorphism. Different colors are used to represent ranges of positive D and D' values. The SNPs are identified by their CNG ID (see additional data file 1). Click here for file
