# Supplementary material for: Comprehensive search for intra- and inter-specific sequence polymorphisms among coding envelope genes of retroviral origin found in the human genome: genes and pseudogenes
Source: BMC Genomics. 2005 Sep 9;6:117. doi: 10.1186/1471-2164-6-117 (PMC1236922; doi:10.1186/1471-2164-6-117)
Supplement: Additional data file 1 — is a table listing the polymorphism of HERV coding envelope genes. [file 1471-2164-6-117-S1.DOC]

Table A. Summary of polymorphism of HERV coding envelope genes

|  | **SNP** | | | | |  | **AA** | | |
| --- | --- | --- | --- | --- | --- | --- | --- | --- | --- |
|  | **nt position** | **CNG ID**^1^ | **A1** | **A2** | **FreqA1** |  | **AA position** | **AA1** | **AA2** |
|  |  |  |  |  |  |  |  |  |  |
| Env Fc(1) | 364 | 29939734 | G | A | 0.28 |  | 122 | Ala | Thr |
| NT_011651.14 | 678 | 29939735 | G | A | 0.94 |  | 226 | Pro | Pro |
|  | 705 | 29939736 | C | T | 0.25 |  | 235 | His | His |
|  | 1202 | 29939737 | C | A | 0.98 |  | 401 | Ala | Asp |
|  | 1278 | 29939738 | C | T | 0.99 |  | 426 | Ala | Ala |
|  | 1644 | 29939739 | C | T | 0.99 |  | 548 | Ser | Ser |
|  | 1707 | 29939740 | C | T | 0.99 |  | 569 | Ser | Ser |
|  |  |  |  |  |  |  |  |  |  |
| Env Fc(2) | 294 | 29939620 | T | C | 0.26 |  | 98 | Cys | Cys |
| NT_007914.13 | 450 | 29939621 | C | T | 0.99 |  | 150 | Ile | Ile |
|  | 525 | 29939622 | C | T | 0.99 |  | 175 | Asp | Asp |
|  | 552 | 29939623 | T | C | 0.66 |  | 184 | Ile | Ile |
|  | 583 | 29939624 | G | A | 0.95 |  | 195 | Gly | Ser |
|  | 653 | 29939626 | C | - | 0.97 |  |  |  |  |
|  | 684 | 29939627 | C | G | 0.99 |  | 228 | Asp | Glu |
|  | 718 | 29939628 | C | T | 0.99 |  | 240 | Leu | Phe |
|  | 737 | 29939629 | C | T | 0.99 |  | 246 | Pro | Leu |
|  | 763 | 29939630 | C | A | 0.89 |  | 255 | Arg | Ser |
|  | 838 | 29939631 | T | C | 0.99 |  | 280 | Cys | Arg |
|  | 1138 | 29939632 | A | G | 0.24 |  | 380 | Ile | Val |
|  |  |  |  |  |  |  |  |  |  |
| Env FRD | 183 | 29939633 | G | A | 0.98 |  | 61 | Ser | Ser |
| NT_007592.13 | 1075 | 29939634 | G | A | 0.98 |  | 359 | Ala | Thr |
|  | 1100 | 29939635 | C | T | 0.99 |  | 367 | Thr | Met |
|  | 1293 | 29939636 | T | C | 0.97 |  | 431 | Cys | Cys |
|  |  |  |  |  |  |  |  |  |  |
| Env H1 | 162 | 29939637 | C | T | 0.99 |  | 54 | Ala | Ala |
| NT_005403.14 | 241 | 29939638 | T | G | 0.50 |  | 81 | Leu | Val |
|  | 450 | 29939639 | T | A | 0.50 |  | 150 | Phe | Leu |
|  | 530 | 29939640 | T | C | 0.98 |  | 177 | Leu | Pro |
|  | 999 | 29939641 | C | T | 0.99 |  | 333 | Leu | Leu |
|  |  |  |  |  |  |  |  |  |  |
| Env H3 | 227 | 29939727 | T | G | 0.98 |  | 76 | Ile | Arg |
| NT_005403.14 | 592 | 29939728 | G | A | 0.03 |  | 198 | Val | Ile |
|  | 901 | 29939729 | A | G | 0.99 |  | 301 | Thr | Ala |
|  | 939 | 29939730 | A | T | 0.98 |  | 313 | Val | Val |
|  | 1471 | 29939731 | G | A | 0.77 |  | 491 | Ala | Thr |
|  |  |  |  |  |  |  |  |  |  |
| Env K1 | 131 | 29939709 | C | T | 0.86 |  | 44 | Ala | Val |
| NT_029419.10 | 398 | 29939710 | A | C | 0.73 |  | 133 | His | Pro |
|  | 800 | 29939711 | G | A | 0.98 |  | 267 | Arg | Gln |
|  | 1208 | 29939712 | C | A | 0.85 |  | 403 | Thr | Asn |
|  | 1344 | 29939713 | C | T | 0.99 |  | 448 | Ile | Ile |
|  | 1445 | 29939714 | T | C | 0.71 |  | 482 | Met | Thr |
|  | 1475 | 29939715 | C | G | 0.96 |  | 492 | Ser | Cys |
|  | 1792 | 29939716 | G | A | 0.99 |  | 598 | Glu | Lys |
|  | 1950 | 29939717 | A | G | 0.00 |  | 650 | Leu | Leu |
|  | 1953 | 29939718 | G | A | 0.72 |  | 651 | Leu | Leu |
|  | 1967 | 29939719 | T | G | 0.00 |  | 656 | Phe | Cys |
|  | 1981 | 29939720 | C | T | 0.99 |  | 661 | Arg | stop |
|  | 1993 | 29939721 | T | G | 0.00 |  | 665 | Tyr | Asp |
|  | 2000 | 29939722 | G | A | 0.99 |  | 667 | Arg | Gln |
|  | 2019 | 29939723 | G | A | 0.72 |  | 673 | Thr | Thr |
|  | 2040/2044 | 29939724 | AAAAG | ----- | 0.72 |  |  |  |  |
|  |  |  |  |  |  |  |  |  |  |
| Env K2 | 43 | 29939934 | C | T | 0.99 |  | 15 | Arg | Trp |
| NT_007819.14 | 58 | 29939935 | C | T | 0.99 |  | 20 | Arg | stop |
|  | 677 | 29939936 | C | G | 0.99 |  | 226 | Pro | Arg |
|  | 778 | 29939937 | G | A | 0.99 |  | 260 | Gly | Arg |
|  | 1035 | 29939938 | C | T | 0.87 |  | 345 | Ala | Ala |
|  | 1316 | 29939939 | T | C | 0.99 |  | 439 | Met | Thr |
|  | 1325 | 29939940 | C | T | 0.95 |  | 442 | Pro | Leu |
|  | 1344 | 29939941 | C | T | 0.99 |  | 448 | Ser | Ser |
|  |  |  |  |  |  |  |  |  |  |
| Env K4 | 170 | 29939651 | C | T | 0.97 |  | 57 | Thr | Met |
| NT_007299.12 | 223 | 29939652 | A | G | 0.99 |  | 75 | Ser | Gly |
|  | 328 | 29939653 | C | T | 0.98 |  | 110 | Pro | Ser |
|  | 483/484 | 29939654 | --- | CCT | 0.27 |  |  |  |  |
|  | 610 | 29939655 | C | T | 0.84 |  | 204 | Arg | Trp |
|  | 685 | 29939656 | G | A | 0.99 |  | 229 | Glu | Lys |
|  | 876 | 29939657 | C | T | 0.84 |  | 292 | Ser | Ser |
|  | 1085 | 29939658 | G | A | 0.99 |  | 362 | Arg | His |
|  | 1106 | 29939659 | A | C | 0.86 |  | 369 | Asp | Ala |
|  | 1144 | 29939660 | G | T | 0.59 |  | 382 | Val | Leu |
|  | 1403 | 29939661 | C | T | 0.88 |  | 468 | Thr | Ile |
|  | 1674 | 29939662 | T | C | 0.99 |  | 558 | Cys | Cys |
|  | 1757 | 29939663 | C | T | 0.87 |  | 586 | Thr | Ile |
|  | 1892 | 29939664 | C | A | 0.99 |  | 631 | Thr | Asn |
|  |  |  |  |  |  |  |  |  |  |
| Env R | 269 | 29939697 | C | T | 0.06 |  | 90 | Thr | Ile |
| NT_007758.10 | 499 | 29939698 | G | C | 0.99 |  | 167 | Asp | His |
|  | 575 | 29939699 | G | A | 0.49 |  | 192 | Cys | Tyr |
|  | 667 | 29939700 | C | T | 0.91 |  | 223 | Arg | stop |
|  | 707 | 29939701 | A | G | 0.99 |  | 236 | Tyr | Cys |
|  | 1442 | 29939702 | A | G | 0.06 |  | 481 | Asn | Ser |
|  | 1696 | 29939703 | G | A | 0.95 |  | 566 | Gly | Arg |
|  | 1706 | 29939704 | A | G | 0.04 |  | 569 | Asn | Ser |
|  | 1749 | 29939705 | C | T | 0.58 |  | 583 | Val | Val |
|  | 1847 | 29939706 | C | T | 0.58 |  | 627 | Ser | Leu |
|  | 1979 | 29939707 | G | A | 0.45 |  | 671 | Arg | Gln |
|  | 1990 | 29939708 | C | T | 0.97 |  | 675 | Arg | *Ter* |
|  |  |  |  |  |  |  |  |  |  |
| Env Rb | 17 | 19432301 | C | T | 0.59 |  | 6 | Thr | Met |
| NT_022517.16 | 93 | 29939690 | T | G | 0.94 |  | 31 | Pro | Pro |
|  | 670 | 29939691 | T | A | 0.92 |  | 224 | Trp | Arg |
|  | 833 | 29939692 | G | A | 0.99 |  | 278 | Trp | stop |
|  | 862 | 19384366 | T | C | 0.15 |  | 288 | Cys | Arg |
|  | 953 | 29939693 | C | G | 0.99 |  | 318 | Thr | Ser |
|  | 962 | 19431429 | T | C | 0.15 |  | 321 | Ile | Thr |
|  | 1099 | 29939694 | A | G | 0.97 |  | 367 | Asn | Asp |
|  | 1126 | 29939695 | G | A | 0.98 |  | 376 | Asp | Asn |
|  | 1311 | 29939696 | G | A | 0.97 |  | 437 | Trp | stop |
|  |  |  |  |  |  |  |  |  |  |
| Env T | 519 | 29939670 | T | C | 0.99 |  | 173 | Ser | Ser |
| NT_011295.10 | 520 | 29939671 | C | T | 0.88 |  | 174 | Arg | Cys |
|  | 524 | 29939672 | T | C | 0.66 |  | 175 | Val | Ala |
|  | 586 | 29939673 | C | G | 0.79 |  | 196 | Pro | Ala |
|  | 592 | 29939674 | G | T | 0.79 |  | 198 | Ala | Ser |
|  | 684 | 29939675 | C | T | 0.98 |  | 228 | Ile | Ile |
|  | 700 | 29939676 | T | C | 0.65 |  | 234 | Ser | Pro |
|  | 705 | 29939677 | - | C | 0.78 |  | 235 |  |  |
|  | 715 | 29939678 | G | A | 0.86 |  | 239 | Gly | Arg |
|  | 785 | 29939679 | T | A | 0.87 |  | 262 | Leu | Gln |
|  | 970 | 29939680 | C | T | 0.79 |  | 324 | Arg | Cys |
|  | 994 | 29939681 | C | T | 0.99 |  | 332 | Arg | stop |
|  | 1000 | 29939682 | C | T | 0.78 |  | 334 | Arg | Cys |
|  | 1053 | 29939683 | C | T | 0.99 |  | 351 | Thr | Thr |
|  | 1112 | 29939684 | C | A | 0.87 |  | 371 | Ser | Tyr |
|  | 1140 | 29939685 | A | G | 0.79 |  | 380 | Ala | Ala |
|  | 1143 | 29939686 | C | T | 0.65 |  | 381 | Pro | Pro |
|  | 1212 | 29939687 | C | A | 0.75 |  | 404 | Leu | Leu |
|  | 1605 | 29939688 | A | G | 0.99 |  | 535 | Gly | Gly |
|  | 1653 | 29939689 | C | T | 0.99 |  | 551 | His | His |
|  |  |  |  |  |  |  |  |  |  |
| Env W | 413 | 29939667 | G | A | 0.95 |  | 138 | Arg | Gln |
| NT_007933.13 | 920 | 29939668 | G | A | 0.84 |  | 307 | Ser | Asn |

^1^ The CNG ID correspond to internal identifiers of the database of the Centre National de Genotypage (CNG). These numbers are unique and not correlated with a position along a reference sequence.
