# Supplementary material for: Comprehensive search for intra- and inter-specific sequence polymorphisms among coding envelope genes of retroviral origin found in the human genome: genes and pseudogenes
Source: BMC Genomics. 2005 Sep 9;6:117. doi: 10.1186/1471-2164-6-117 (PMC1236922; doi:10.1186/1471-2164-6-117)
Supplement: Additional data file 2 — is a figure showing polymorphism combinations and estimated frequencies for haplotypes in the 12 HERV coding env genes. The SNPs are identified by their CNG ID (see additional data file 1). [file 1471-2164-6-117-S2.PDF]

ENV_R													
	Freq	29939697	29939698	29939699	29939700	29939701	29939702	29939703	29939704	29939705	29939706	29939707	29939708
H1	0.386	T	G	G	C	A	G	G	G	T	T	G	C
H2	0.373	T	G	A	C	A	G	G	G	C	C	A	C
H3	0.096	T	G	A	T	A	G	G	G	C	C	A	C
H4	0.049	C	G	G	C	A	A	G	A	C	C	G	C
H5	0.047	T	G	A	C	A	G	A	G	C	C	A	C
H6	0.024	T	G	A	C	A	G	G	G	C	C	A	T
H7	0.008	C	G	G	C	A	A	A	A	C	C	G	C
H8	0.008	T	G	G	C	A	G	G	A	T	T	G	C
H9	0.008	T	G	G	C	A	G	G	G	C	T	G	C
H10	0.001	C	G	G	T	A	A	G	A	C	C	G	C

ENV_H1						
	Freq	29939637	29939638	29939639	29939640	29939641
H1	0.492	C	G	T	T	C
H2	0.468	C	T	A	T	C
H3	0.017	C	T	A	C	C
H4	0.012	T	T	A	T	C
H5	0.011	C	T	T	T	C

ENV_R(b)											
	Freq	19432301	29939690	29939691	29939692	19384366	29939693	19431429	29939694	29939695	29939696
H1	0.396	T	T	T	G	C	C	C	A	G	G
H2	0.317	C	T	T	G	C	C	C	A	G	G
H3	0.155	C	T	T	G	T	C	T	A	G	G
H4	0.056	C	G	A	G	C	C	C	A	G	G
H5	0.029	C	T	A	G	C	C	C	A	G	G
H6	0.027	C	T	T	G	C	C	C	G	G	A
H7	0.017	T	T	T	G	C	C	C	A	A	G

ENV_K2									
	Freq	29939934	29939935	29939936	29939937	29939938	29939939	29939940	29939941
H1	0.805	C	C	C	G	C	T	C	C
H2	0.129	C	C	C	G	T	T	C	C
H3	0.049	C	C	C	G	C	T	T	C
H4	0.012	C	C	C	G	C	T	C	T
H5	0.005	C	C	C	G	T	T	T	C

ENV_T																					
	Freq	29939670	29939671	29939672	29939673	29939674	29939675	29939676	29939677	29939678	29939679	29939680	29939681	29939682	29939683	29939684	29939685	29939686	29939687	29939688	29939689
H1	0.614	T	C	T	C	G	C	T	-	G	T	C	C	C	C	C	A	C	C	A	C
H2	0.199	T	C	C	G	T	C	C	C	G	T	T	C	T	C	C	G	T	A	A	C
H3	0.112	T	T	C	C	G	C	C	-	A	A	C	C	C	C	A	A	T	C	A	C
H4	0.023	T	C	T	C	G	C	T	-	G	T	C	T	C	C	C	A	C	C	A	C
H5	0.018	T	C	T	C	G	T	T	-	G	T	C	C	C	C	C	A	C	A	A	C
H6	0.007	T	C	C	C	G	C	T	C	G	T	C	C	C	C	C	A	C	C	A	C
H7	0.007	T	C	C	C	G	T	T	C	G	T	C	C	C	C	C	A	C	C	A	C
H8	0.006	T	C	C	G	T	T	C	C	G	T	T	C	T	C	C	G	T	A	A	C
H9	0.005	T	C	T	C	G	T	T	-	G	T	C	C	C	C	C	A	C	C	A	C
H10	0.004	T	T	C	C	G	C	C	-	A	A	C	T	C	C	A	A	T	C	A	C
H11	0.002	T	C	C	G	T	C	C	-	G	A	C	C	T	C	C	A	T	A	A	C
H12	0.002	T	C	C	G	T	C	C	-	A	A	C	C	T	C	C	A	T	A	A	C
H13	0.002	T	C	C	G	T	C	C	-	G	T	C	C	T	C	C	A	T	A	A	C
H14	0.002	T	C	C	G	T	C	C	-	A	T	C	C	T	C	C	A	T	A	A	C

ENVK1																	
	Freq	29939709	29939710	29939711	29939712	29939713	29939714	29939715	29939716	29939717	29939718	29939719	29939720	29939721	29939722	29939723	29939724
H1	0.547	C	A	G	C	C	T	C	G	G	G	G	C	G	G	G	AAAAG
H2	0.267	C	C	G	C	C	C	C	G	G	A	G	C	G	G	A	-----
H3	0.110	T	A	G	A	C	T	C	G	G	G	G	C	G	G	G	AAAAG
H4	0.034	T	A	G	A	C	T	G	G	G	G	G	C	G	G	G	AAAAG
H5	0.014	C	A	A	C	C	T	C	G	G	G	G	C	G	G	G	AAAAG
H6	0.014	C	A	G	C	C	T	C	G	G	G	G	C	G	A	G	AAAAG
H7	0.014	C	C	G	C	T	C	C	G	G	A	G	C	G	G	A	-----

ENV_H3						
	Freq	29939727	29939728	29939729	29939730	29939731
H1	0.708	T	A	A	A	G
H2	0.220	T	A	A	A	A
H3	0.029	T	G	A	A	G
H4	0.023	G	A	A	A	G
H5	0.010	T	A	A	T	G
H6	0.005	T	G	A	T	G
H7	0.003	T	A	A	T	A

ENV_K4															
	Freq	29939651	29939652	29939653	29939654	29939655	29939656	29939657	29939658	29939659	29939660	29939661	29939662	29939663	29939664
H1	0.385	C	A	C	CCT	C	G	C	G	A	T	C	T	C	C
H2	0.278	C	A	C	---	C	G	C	G	A	G	C	T	C	C
H3	0.136	C	A	C	CCT	C	G	C	G	C	G	T	T	T	C
H4	0.134	C	A	C	CCT	T	G	T	G	A	G	C	T	C	C
H5	0.018	T	A	C	CCT	C	G	C	G	A	T	C	T	C	C
H6	0.013	C	A	C	CCT	C	G	C	G	A	G	C	T	C	C
H7	0.012	T	A	C	CCT	C	G	C	G	A	T	C	T	C	A
H8	0.012	C	A	T	CCT	T	G	T	G	A	G	C	T	C	C
H9	0.012	C	A	T	CCT	T	G	T	G	A	G	C	C	C	C

ENV_F(c)1								
	Freq	29939734	29939735	29939736	29939737	29939738	29939739	29939740
H1	0.658	A	G	T	C	C	C	C
H2	0.229	G	G	C	C	C	C	C
H3	0.054	A	A	T	C	C	C	C
H4	0.018	G	G	T	A	C	C	C
H5	0.017	G	G	T	C	C	C	C
H6	0.012	G	G	C	C	T	C	C
H7	0.012	G	G	C	C	C	T	C

ENV_F(c)2													
	Freq	29939620	29939621	29939622	29939623	29939624	29939626	29939627	29939628	29939629	29939630	29939631	29939632
H1	0.419	C	C	C	T	G	C	C	C	C	C	T	G
H2	0.266	C	C	C	C	G	C	C	C	C	C	T	G
H3	0.116	T	C	C	T	G	C	C	C	C	C	T	A
H4	0.075	T	C	C	T	G	C	C	C	C	A	T	A
H5	0.042	C	C	C	C	A	C	C	C	C	C	T	G
H6	0.028	T	C	C	T	G	-	C	C	C	A	T	A
H7	0.014	T	C	C	T	G	C	C	C	C	C	T	G
H8	0.012	C	C	C	C	G	C	C	C	C	C	C	G
H9	0.008	C	C	C	C	G	C	G	C	C	C	T	G
H10	0.007	C	C	C	T	A	C	C	C	C	C	T	G
H11	0.006	C	C	C	C	G	C	C	C	C	C	T	A
H12	0.004	T	C	C	C	A	C	C	C	C	A	T	A
H13	0.003	T	C	C	C	G	C	G	C	C	C	T	A

ENV_FRD					
	Freq	29939633	29939634	29939635	29939636
H1	0.958	G	G	C	T
H2	0.027	A	G	C	C
H3	0.015	G	A	C	T

ENV_W			
	Freq	29939667	29939668
H1	0.844	G	G
H2	0.108	G	A
H3	0.048	A	A
