# Supplementary figures and images for: Comprehensive search for intra- and inter-specific sequence polymorphisms among coding envelope genes of retroviral origin found in the human genome: genes and pseudogenes
Source: BMC Genomics. 2005 Sep 9;6:117. doi: 10.1186/1471-2164-6-117 (PMC1236922; doi:10.1186/1471-2164-6-117)

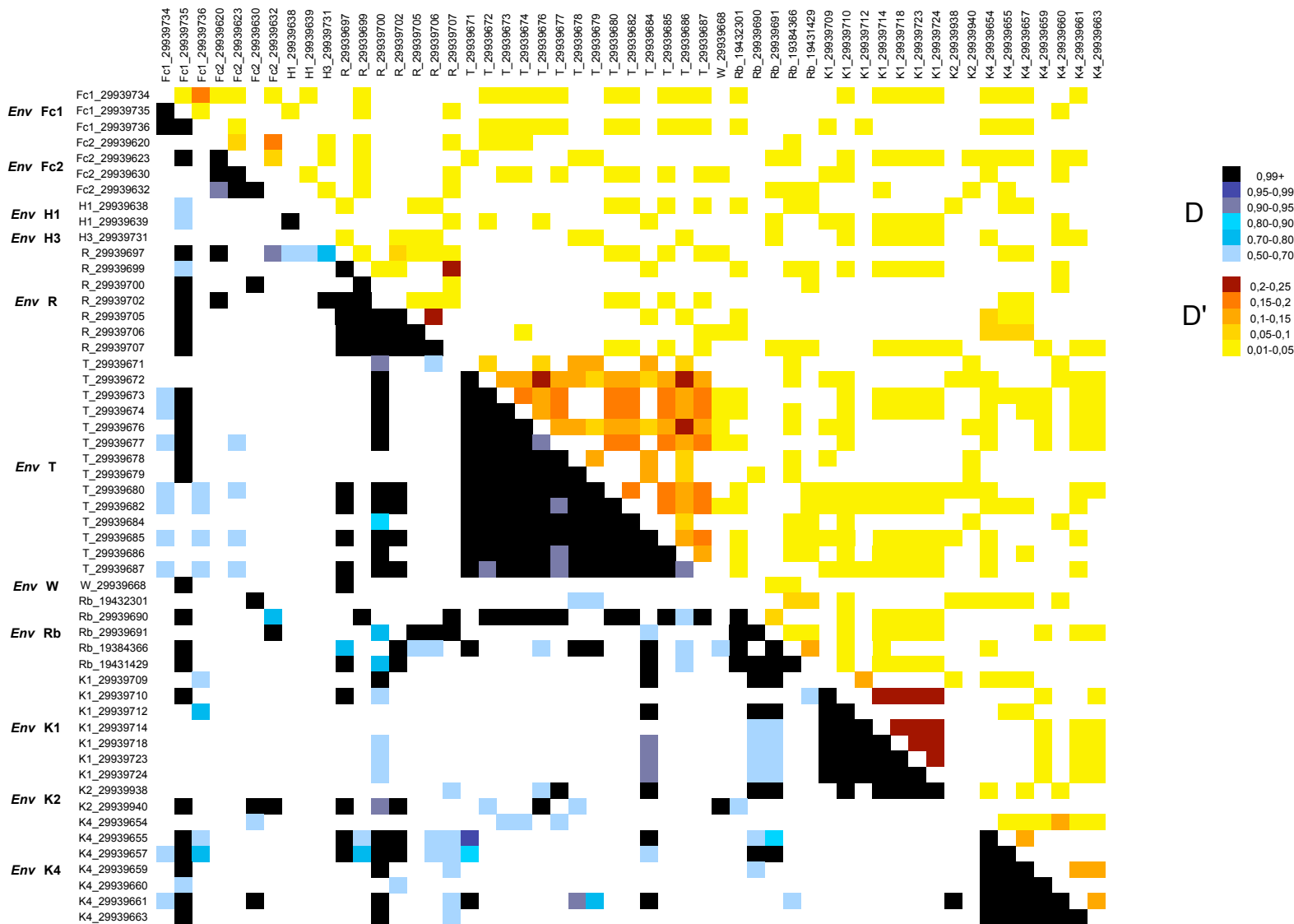

Supplement: Additional data file 3 — is a figure showing the LD plot of 12 HERV coding env genes. The LD pattern is shown with the D values above and the D' values below the diagonal, and estimated allele frequencies for each polymorphism. Different colors are used to represent ranges of positive D and D' values. The SNPs are identified by their CNG ID (see additional data file 1). [file 1471-2164-6-117-S3.PDF]
